# Temperature-Dependent Reverse-Recovery Behavior Analysis and Circuit-Level Mitigation of Superjunction MOSFETs

**DOI:** 10.3390/mi16111252

**Published:** 2025-10-31

**Authors:** Wenrong Cui, Peng Liao, Yanghao Wang, Jianbin Guo, Yafen Yang, David Wei Zhang, Hang Xu

**Affiliations:** State Key Laboratory of Integrated Chips and Systems, School of Microelectronics, Fudan University, Shanghai 200433, China; 22112020081@m.fudan.edu.cn (W.C.); yafen_yang@fudan.edu.cn (Y.Y.);

**Keywords:** superjunction MOSFETs, reverse recovery, temperature-dependent, TCAD

## Abstract

This study explores the temperature dependence of reverse-recovery behavior in superjunction metal-oxide-semiconductor field-effect-transistors (MOSFETs) using experiments and Technology Computer-Aided Design (TCAD) simulations. Results show that at 145 °C, switching failure occurs due to severe reverse-recovery degradation. The main cause is the temperature-induced increase in carrier lifetime, leading to a higher reverse-recovery charge, current, and time. A practical solution is proposed by adding a small parallel capacitor, which effectively suppresses reverse recovery and improves switching reliability. This work provides physical insight and a simple strategy for optimizing superjunction MOSFET performance in high-temperature power electronics.

## 1. Introduction

Superjunction MOSFETs (SJ-MOSFETs), featuring alternating deep-trench P-type and N-type pillar drift regions, utilize the principle of charge balance to surpass the traditional “silicon limit” in the trade-off between specific on-resistance (*Ron,sp*) and breakdown voltage (*BV*) observed in conventional VDMOS devices [1,2]. Compared with traditional silicon-based power MOSFETs, SJ-MOSFETs offer a significantly improved compromise between blocking capability and conduction loss, making them highly suitable for 500–900 V power devices used in high-frequency industrial applications [3,4,5,6].

However, the unique superjunction structure inherently leads to poor reverse-recovery characteristics [7,8,9], which has attracted considerable research attention [10,11,12]. Several structural innovations have been proposed to mitigate this limitation. For instance, incorporating an auxiliary layer between the superjunction region and the N-type substrate can supply additional carriers once the superjunction is fully depleted, thereby enabling a smoother decay of the reverse-recovery current [13]. Forming Schottky contacts on the drain-side P-pillars effectively reduces the total carrier concentration and enhances the reverse-recovery performance [14]. Furthermore, introducing a thin oxide layer between the tunnel diode and the P-/N-pillars suppresses electron injection from the N-pillar region and the N+ substrate into the P-pillar, thereby reducing the reverse-recovery charge (*Qrr*) [15]. In addition to structural optimization, carrier lifetime control—through techniques such as electron, proton, or helium ion irradiation and heavy-metal doping—remains one of the most effective approaches for *Qrr* reduction [16,17], underscoring the critical role of carrier lifetime in reverse-recovery behavior. Moreover, package-level optimizations, such as improved pin layouts, can further suppress reverse-recovery oscillations [18].

In this work, the reverse-recovery characteristics of SJ-MOSFETs are experimentally investigated under various temperature conditions. The temperature dependence of reverse-recovery behavior is analyzed, and device failure due to breakdown during reverse recovery is observed at 145 °C. To further elucidate the underlying physical mechanisms, a TCAD model of the superjunction MOSFET is developed. Simulation results confirm that reverse-recovery performance can be significantly enhanced through circuit-level optimization.

**Figure 1:**
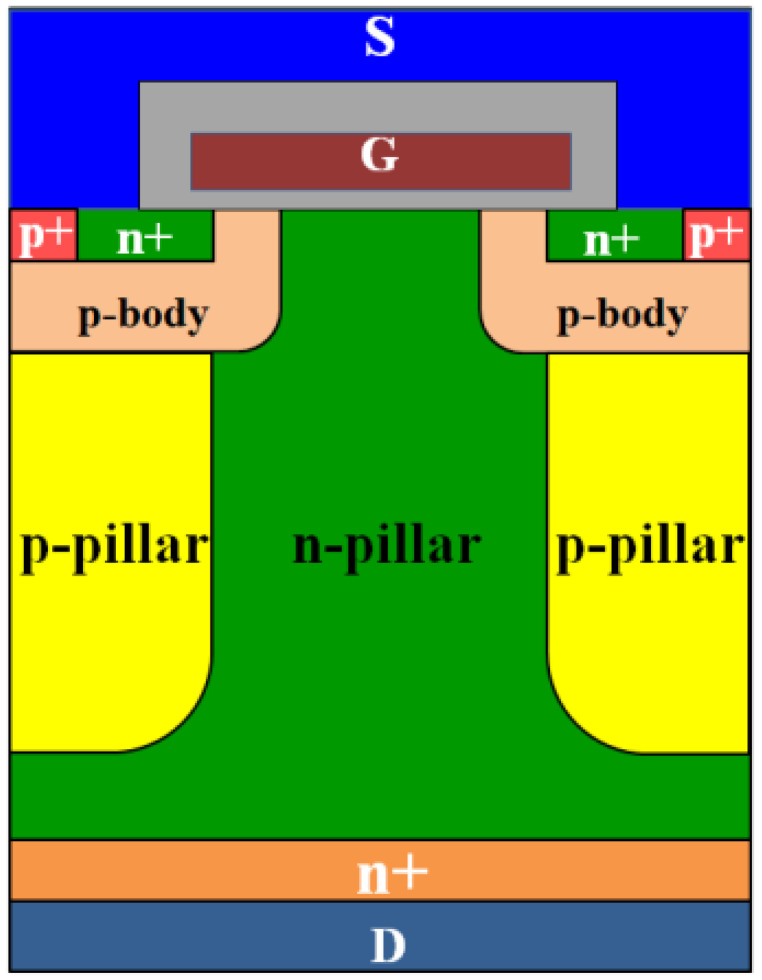
Schematic diagram of cell structure of superjunction MOSFET.

The investigated devices are self-developed 650 V class SJ MOSFETs fabricated using a deep-trench etching and epitaxial regrowth process. The overall device structure is illustrated in Figure 1. The superjunction is realized by deep trench etching followed by selective epitaxial growth to fill the trenches with boron-doped single-crystalline silicon, forming P-pillars. Subsequently, an additional shallow n-type layer was formed on top of the N-pillars to further reduce the specific on-resistance. The structural parameters of the simulated device are as follows: the P-pillar depth is 43 μm, the doping concentration of the P-pillar is 6.2 × 10^15^ cm^−3^, the N-pillar doping concentration is 3.24 × 10^15^ cm^−3^, and the cell pitch is 4 μm.

## 2. Experimental Measurements

**Figure 2:**
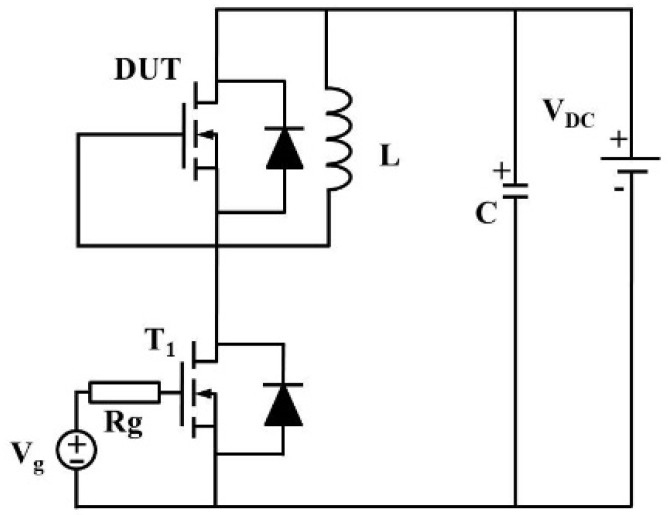
Half-bridge circuit for reverse-recovery testing of superjunction devices. The upper transistor acts as the DUT for reverse-recovery characterization, while the lower transistor serves as the commutation switch.

The double-pulse switching test of the superjunction MOSFET was conducted using a half-bridge circuit, as illustrated in Figure 2. In the test configuration, a large inductance *L* = 350 μH was employed to maintain an approximately constant current throughout the switching cycle. *R_g_* denotes the external gate resistance of the switching transistor *T*_1_. By shorting the gate and source terminals, the intrinsic body diode *D*_2_ of the device under test (DUT) serves as the freewheeling diode. Both transistors used in the half-bridge configuration are identical 650 V SJ MOSFETs. The upper device acts as DUT for reverse-recovery characterization, while the lower one serves as the commutation switch. A computer-controlled function generator provides the pulse signal to the gate driver, generating the gate drive voltage *V_gg_* for *T*_1_. The high-voltage DC source *V_DC_* is connected in parallel with a capacitor *C* to stabilize the supply voltage. In the experiments, *V_DC_* was set to 350 V and *C* to 2.1 μF.

**Figure 3:**
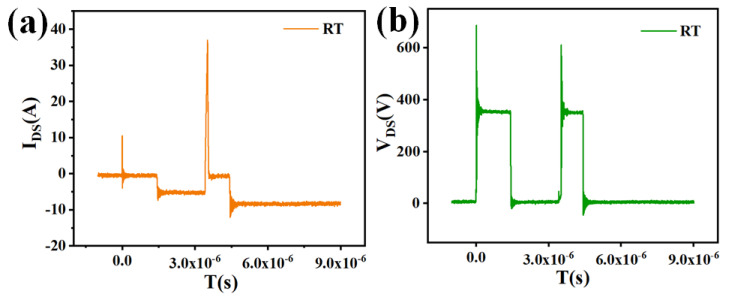
*I_DS_* (**a**) and *V_DS_* (**b**) test results of superjunction MOSFETs.

Figure 3 shows the drain current (*I_D_*_2_) and drain voltage (*V_D_*_2_) waveforms of the SJ-MOSFET under test, where *V_DC_* = 350 V and *R_g_* = 5.1 Ω. Initially, the circuit current is zero. During the first stage, a high-level gate pulse turns on *T_1_*, forming a current loop through the inductor *L*, *T*_1_, and the DC source. In the second stage, the gate voltage is driven low, turning *T*_1_ off. The energy stored in *L* is then released and charges the DUT. The body diode *D*_2_ provides freewheeling current protection, preventing device damage. In the third stage, a high-level pulse is again applied, switching off the DUT. At turn-off, the external voltage across the DUT rapidly reverses polarity, causing the body diode to transition from a forward-biased state to a reverse-blocking state. The forward current does not immediately reverse but decays gradually at a fixed di/dt. When the reverse current reaches its peak value (*I_rrm_*), the body diode voltage also peaks. As the stored charge *Q_rr_* in the drift region during forward conduction is insufficient to sustain further current flow, *I_rrm_* decreases and eventually returns to zero, completing the reverse-recovery process of the SJ-MOSFET.

**Figure 4:**
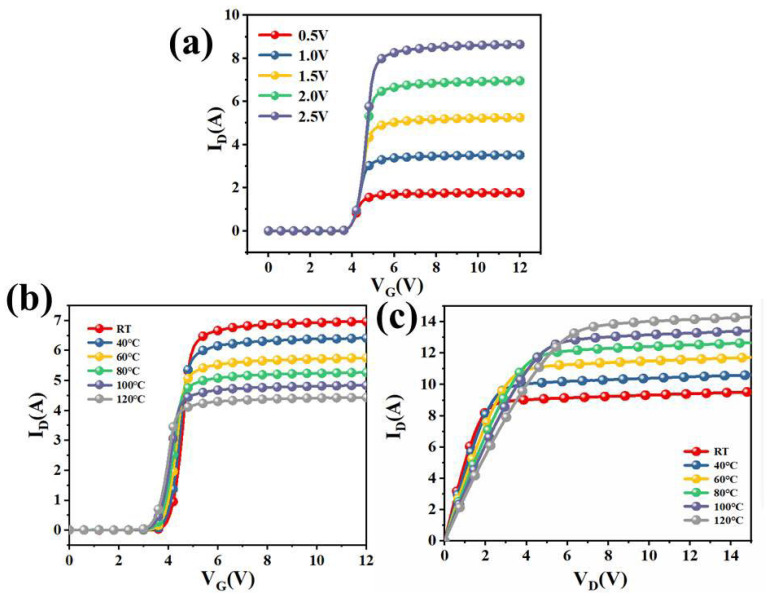
(**a**) Transfer characteristics of superjunction MOSFETs at different *V_D_*. (**b**) Transfer characteristics of superjunction MOSFETs for different temperatures at *V_D_* = 2 V. (**c**) Output characteristics of superjunction MOSFETs for different temperatures at *V_G_* = 5 V.

Self-heating is a common phenomenon during device operation and can significantly affect electrical characteristics. As shown in Figure 4b,c, rising temperature causes noticeable shifts in both the transfer and output characteristics of the device.

**Figure 5:**
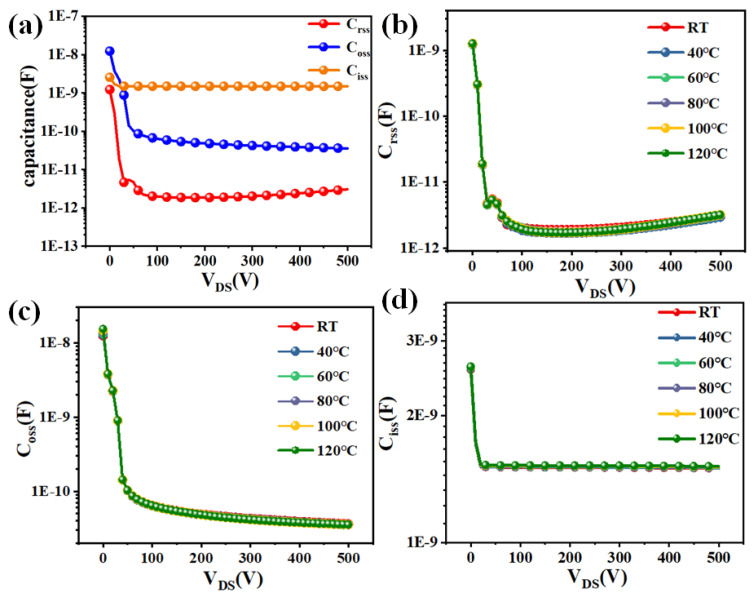
(**a**) *C_rss_*, *C_oss_*, and *C_iss_* of Superjunction MOSFET. (**b**) *C_rss_*, (**c**) *C_oss_*, and (**d**) *C_iss_* at different temperatures.

The capacitance characteristics of SJ-MOSFETs were also investigated. Figure 5a shows the variations of *C_rss_*, *C_oss_*, and *C_iss_* as functions of the drain voltage *V_D_*. Their temperature dependence is presented in Figure 5b–d. Although all three capacitances exhibit slight changes with increasing junction temperature, the variations are minimal, indicating that temperature has a negligible effect on the device’s capacitance behavior.

**Figure 6:**
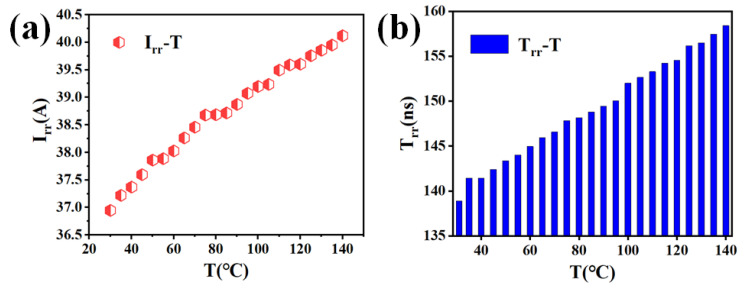
(**a**) Superjunction MOSFET reverse-recovery current as a function of temperature. (**b**) Superjunction MOSFET reverse-recovery time as a function of temperature.

In contrast, temperature has a significant influence on the reverse-recovery characteristics, as illustrated in Figure 6. Both the reverse-recovery current and reverse-recovery time increase with rising temperature, indicating a more difficult device turn-off. When the junction temperature increases from 30 °C to 140 °C, the peak reverse-recovery current rises from 36.97 A to 40.12 A. Similarly, the reverse-recovery time extends by approximately 20 ns.

**Figure 7:**
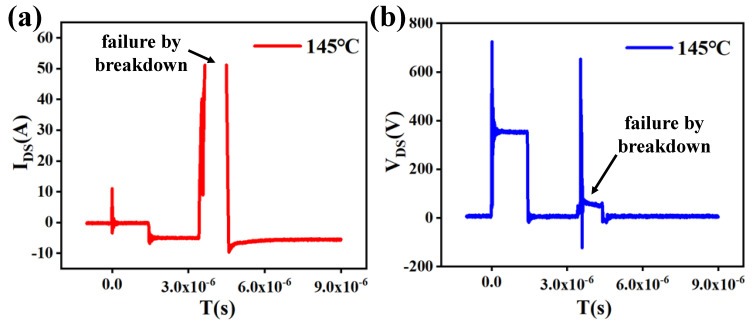
*I_DS_* (**a**) and *V_DS_* (**b**) of reverse-recovery test for superjunction MOSFET at 145 °C.

Moreover, the elevated reverse-recovery current increases the risk of device breakdown. As shown in Figure 7, when the junction temperature reaches 145 °C, the device undergoes breakdown during the reverse-recovery process, leading to switching failure. These results highlight the importance of investigating the temperature dependence of reverse-recovery behavior to ensure the reliable switching performance and operational safety of superjunction MOSFETs.

## 3. Simulation Results and Discussion

**Figure 8:**
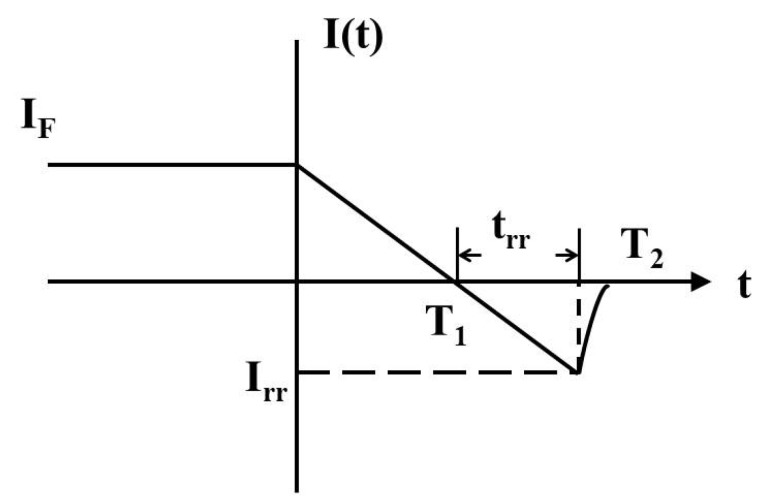
Schematic diagram of the reverse-recovery process.

The reverse-recovery process of the superjunction MOSFET is illustrated in Figure 8. For a given device, an increase in reverse-recovery charge results in a corresponding rise in both the peak reverse-recovery current and the reverse-recovery time. During the interval 0 *< t ≤ T*_2_, the external voltage across the device reverses polarity, causing the current to decrease linearly, as expressed in Equation (1):

(1) $$I(t)=I_{F}-Rt$$

where *R* represents the current decay rate, and *I_F_* stands for forward current. To qualitatively understand the reverse-recovery process and the physical meaning of *R*, it is simplified to a constant.

Thus, the peak reverse-recovery current is closely related to the reverse-recovery time of the device. The total reverse-recovery charge can be expressed as follows [19]:

(2) $$Q(t)=Q(0)\left[\frac{\tau }{T_{1}}\left(1-e^{\frac{-t}{\tau }}\right)-\frac{t}{T_{1}}+1\right]$$

where *Q*(0) represents the reverse-recovery charge in the initial state, *Q*(*t*) represents the remaining reverse-recovery charge after *t* time, and *τ* represents the carrier lifetime. In the above equation, the temperature dependence of *τ* is modeled by the power law [20,21]:

(3) $$\tau =\tau_{0}{\left(\frac{T}{300K}\right)}^{\alpha }$$

where *τ_0_* is the carrier lifetime when the lattice temperature is 300 K, and *T* is the lattice temperature. *α* is the temperature coefficient that quantifies the sensitivity of lifetime to temperature variation, and the typical value of *α* for the Si-base device is 2.1 [22].

**Figure 9:**
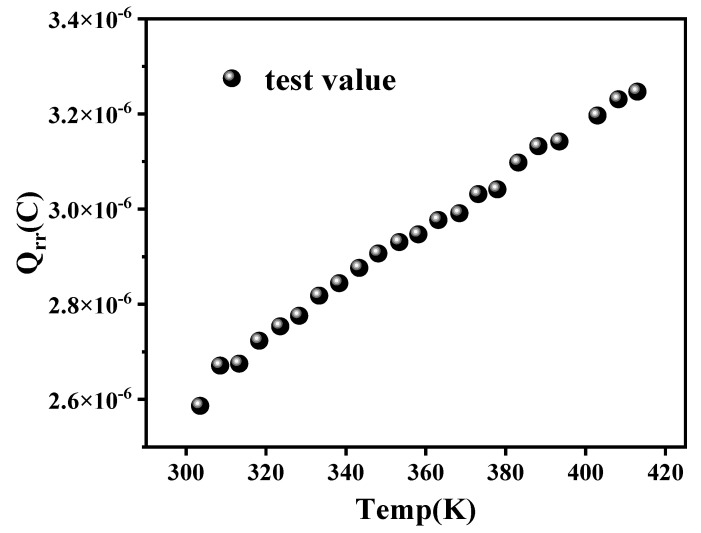
Variations in reverse recovery charge with the increase in temperature.

From Equation (3), it is evident that the carrier lifetime *τ* increases with temperature. As a result, the reverse-recovery charge *Q_rr_* also increases, as shown in Figure 9, leading to corresponding increases in both reverse-recovery time and peak reverse-recovery current (see Figure 6).

**Figure 10:**
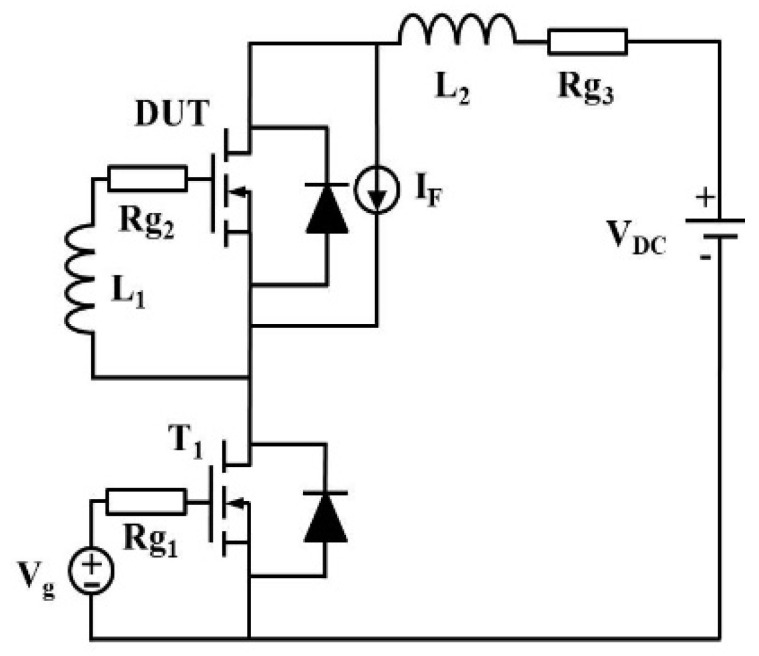
Superjunction MOSFET simulation circuit diagram.

To further verify that the temperature dependence of reverse-recovery behavior is primarily governed by changes in carrier lifetime, TCAD simulations were performed. A reverse-recovery test model was constructed in Sentaurus TCAD, and the corresponding simulation circuit is shown in Figure 10. In the model, *R_g_*_1_ and *R_g_*_2_ represent the total gate resistance, set to 4 Ω and 5 Ω, respectively. *L_1_* denotes the total gate inductance, which includes both the package stray inductance and the external gate loop inductance, with a value of 20 nH. *R_g_*_3_ and *L*_2_ correspond to the stray resistance and inductance of the power loop, set to 0.9 Ω and 10 nH, respectively.

**Figure 11:**
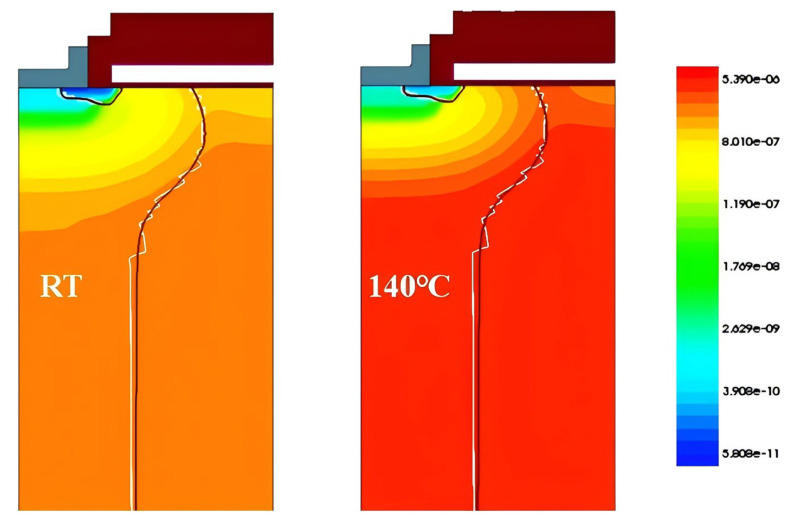
The carrier lifetime distribution of the superjunction MOSFET at RT and 140 °C, respectively.

The simulations were performed using parameters obtained from analytical model fitting. Both device-level and mixed-mode simulations were carried out, incorporating key physical models, such as Auger recombination, avalanche generation, Shockley–Read–Hall (SRH) recombination, high-field mobility saturation, doping-dependent mobility, and bandgap-narrowing effects [23]. The simulated carrier lifetime distributions at different temperatures are shown in Figure 11. A clear increase in carrier lifetime at 140 °C is observed compared to 30 °C.

**Figure 12:**
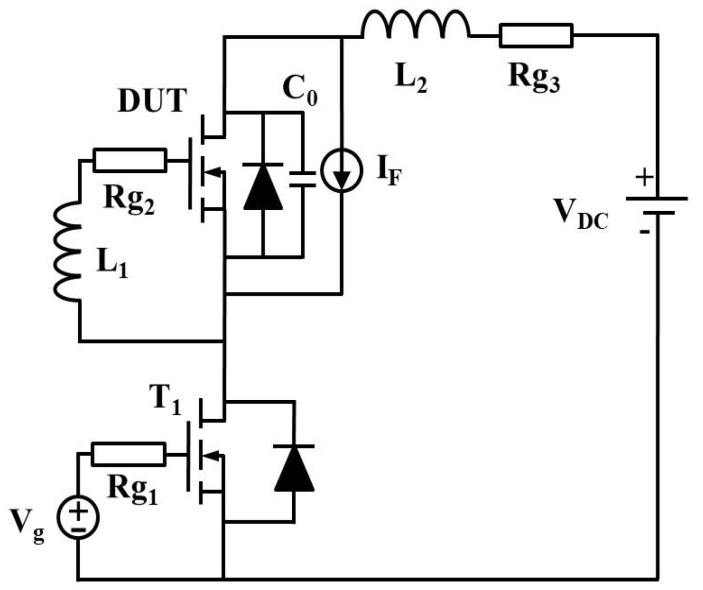
Superjunction MOSFET improved simulation circuit diagram.

To mitigate the degradation of the reverse-recovery performance at elevated temperatures, a capacitor (*C*_0_ = 1.2 nF) was connected in parallel with the DUT, as illustrated in Figure 12. This snubber capacitor provides an alternative current path during the reverse-recovery transition, thereby reducing the peak reverse current and shortening the recovery time.

**Figure 13:**
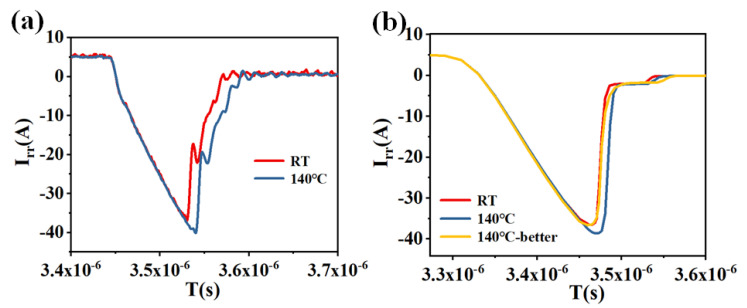
(**a**) Test results of reverse-recovery characteristics. (**b**) Simulation results of reverse recovery at 30 °C, 140 °C, and 140 °C with parallel capacitors.

The effectiveness of this approach was verified through both experimental measurements and TCAD simulations. Figure 13a shows the test results of the reverse-recovery characteristics of the device at 30 °C and 140 °C, and Figure 13b shows the simulation results at the corresponding temperatures. The results show that the inclusion of the parallel snubber capacitor significantly improves the reverse-recovery behavior at elevated temperatures, as indicated by the yellow trace. These results confirm that parallel snubber capacitors provide an effective means of suppressing the temperature-induced degradation of reverse-recovery performance in superjunction MOSFETs.

In this work, a 1.2 nF capacitor was connected in parallel between the drain and source terminals to demonstrate the suppression of reverse-recovery degradation. The value was mainly limited by available laboratory components and the test circuit layout.

Physically, the added capacitor increases the effective *C_DS_*, which is a part of *C_oss_*. An increased *C_DS_* raises the charge required for *V_DS_* transition, thereby reducing the voltage slew rate (*dV/dt*). This delayed voltage transition prolongs the overlap between drain current and voltage waveforms, leading to higher switching loss and reduced efficiency, particularly in high-frequency operation. Therefore, the capacitance value cannot be made too large despite its benefit in reducing reverse-recovery charge. The 1.2 nF capacitor used here represents a practical and moderate value that effectively demonstrates the mitigation mechanism without causing excessive switching-speed degradation. The quantitative dependence of reverse-recovery and switching losses on the capacitor value will be investigated in future studies.

## 4. Conclusions

In this work, the temperature dependence of the reverse-recovery characteristics of superjunction MOSFETs was systematically investigated through a combination of experimental measurements and TCAD simulations. The key findings can be summarized as follows:

1. When the junction temperature reaches 145 °C, device failure occurs during the switching process, indicating that high temperatures can severely compromise the safe operating range and switching reliability of superjunction MOSFETs.
2. The variation in carrier lifetime with temperature is identified as the primary mechanism responsible for the degradation of reverse-recovery performance. As temperature increases, the carrier lifetime extends, leading to increased reverse-recovery charge, higher peak reverse current, and longer reverse-recovery time.
3. The reverse-recovery performance of superjunction MOSFETs can be effectively improved by connecting a small snubber capacitor in parallel with the device. This modification reduces the reverse-recovery current, reverse-recovery charge, and reverse-recovery time, thereby enhancing device reliability under high-temperature operation.

Although the proposed mitigation strategy is implemented externally as a small parallel capacitor, it originates from a physical understanding of the temperature-dependent reverse-recovery mechanism in superjunction MOSFETs. Specifically, the degradation arises from enhanced carrier lifetime and excess stored charge in the drift region at elevated temperature, which can be counteracted by providing an additional charge-balancing path. Thus, this study bridges the physical mechanism and the practical mitigation strategy, offering insight that is relevant to both device design and circuit implementation.

## Data Availability

The original contributions presented in this study are included in the article. Further inquiries can be directed to the corresponding author.
